# Conceptual Issues Concerning Internet Addiction and Internet Gaming Disorder: Further Critique on Ryding and Kaye (2017)

**DOI:** 10.1007/s11469-017-9818-z

**Published:** 2017-10-17

**Authors:** Mark D. Griffiths

**Affiliations:** International Gaming Research Unit, Psychology Department, Nottingham Trent University, 50 Shakespeare Street, Nottingham, NG1 4FQ UK

**Keywords:** Internet addiction, Internet Gaming Disorder, Gaming addiction, Online addictions, Problem gaming, Problem gambling

## Abstract

The recent commentary paper by Ryding and Kaye *Journal of Mental Health and Addictio*n (doi 10.1007/s11469-017-9811-6, 2017) rightly claimed that “internet addiction” (IA) is a conceptual minefield and raised some important issues for researchers and treatment providers working in the online addiction field. In the present commentary paper, some of the assertions made by Ryding and Kaye are briefly critiqued and extended. More specifically, the present paper (i) examines IA and Internet-based addictions and argues that IA is now a misnomer, (ii) examines IA and its relationship to Internet Gaming Disorder (IGD) and argues IA and IGD are two completely different constructs, and that IGD is a sub-type of gaming disorder rather than a sub-type of IA, (iii) argues that the time spent engaging in online activities is not a good criterion for assessing online addictions and that the context of use is far more important criterion, and (iv) argues that those researchers working in the IA field can learn a lot from the problem gambling field in collecting robust data. More specifically, one of the innovative ways forward could be to build strategic partnerships with commercial online companies to analyze their behavioral tracking data.

## Internet Addiction vs. Internet-Based Addictions: Some Further Thoughts

The recent commentary paper by Ryding and Kaye (2017) on the concept of Internet addiction (IA) raised a number of theoretical issues that anyone working in the field would recognize as important, and I wholeheartedly agree that the area is (and always has been) a “conceptual minefield.” In this commentary, I briefly critique and extend some of the assertions made by Ryding and Kaye.

### Internet Addiction Is Now a Misnomer

Ryding and Kaye began their paper by noting that IA has only “recently been identified as being addictive” (p.1) but then cite references from over 20 years ago on the topic including one of my own (i.e., Griffiths 1996a). In 1995 and 1996, I published a number of papers and articles on “technological addictions” and IA (Griffiths 1995a, b, 1996a, b), all arguing that addictions *to* and *on* the Internet were likely to be major areas of concern in the addictive behavior field. In these papers and my other early writings in the field (Griffiths 1998, 1999, 2000), I constantly made the point that there was a difference between what we now term “generalized internet addiction” (i.e., where an individual spends almost all of their waking time online engaged in more than one online activity to the neglect of all other important activities in their life) and “specific internet addictions” (i.e., where an individual spends almost all of their waking time engaged in just online activity, such as gambling or gaming, to the neglect of all other important activities in their life).

Since my writings more than two decades ago, I have come to the view that “internet addiction” and (similarly) “smartphone addiction” (also highlighted by Ryder and Kaye) are misnomers. Internet addicts and smartphone addicts are no more addicted to the Internet or their smartphones than alcoholics are addicted to bottles (Griffiths and Kuss 2017; Griffiths and Pontes 2014; Kim and Kim 2010). Individuals addicted to online gaming, online gambling, online sex, or online shopping are not Internet addicts. They are gambling addicts, gaming addicts, sex addicts, or shopping addicts that are using the medium of the Internet to engage in their addictive behavior. There are of course some activities—such as social networking—that could be argued to be a genuine and “pure” type of “internet addiction” because the activity of social networking can only take place online and there is no offline equivalent. However, the addiction is to an application rather than the Internet itself, and this should be termed “social networking addiction” rather than a type or sub-type of IA.

### Internet Addiction and Internet Gaming Disorder Are Two Completely Different Constructs

Ryding and Kaye assert that much “of the recent literature in the realm of IA has focused upon Internet Gaming Disorder (IGD)” (p.3). However, IA and IGD are two completely different constructs as have been shown empirically in a largescale nationally representative study (i.e., Király et al. 2014) and argued theoretically (Griffiths and Pontes 2014). The conflation of these two independent constructs has not been helped by the accompanying text for IGD in the latest (fifth) edition of the *Diagnostic and Statistical Manual of Mental Disorders* (DSM-5; American Psychiatric Association 2013). More specifically, the DSM-5 asserts that:

*There are no well-researched subtypes for Internet gaming disorder to date. Internet gaming disorder most often involves specific Internet games, but it could involve non-Internet computerized games as well, although these have been less researched. It is likely that preferred games will vary over time as new games are developed and popularized, and it is unclear if behaviors and consequence associated with Internet gaming disorder vary by game type…Internet gaming disorder has significant public health importance, and additional research may eventually lead to evidence that Internet gaming disorder (also commonly referred to as Internet use disorder, Internet addiction, or gaming addiction) has merit as an independent disorder *(p.796).

Not only do these statements conflate IGD with IA but also claim that IGD can (confusingly) include offline gaming disorder, a situation that my colleagues and I recently described as leading to *“chaos and confusion”* in the online addiction field (Kuss et al. 2017; p.103). Personally, I would argue that IGD is a sub-type of gaming disorder rather than a sub-type of IA. Ryding and Kaye appear to implicitly agree with this because their paper also asserts:

*One means by which researchers could move forward in this regard is to establish the validity of IGD to a wider range of gaming formats…Arguably, there are a range of forms of “online” gaming, including social networking site (SNS) games which are Internet-mediated and thus by definition, would appear under the remit of IGD…given these are often played on mobile devices rather than on more traditional PC or console platforms *(p.3).

Even before the advent of online gaming in the early 2000s, I argued that there were many different platforms on which individuals could play videogames including gaming machines in amusement arcades, dedicated home consoles (e.g., *Sony PlayStation*), personal computers, and handheld devices (e.g., *Nintendo GameBoy*) (Griffiths 1991, 1993). None of these gaming platforms involved being online, yet gaming on all of these platforms can be potentially addictive for a minority that play videogames on them (Griffiths et al. 2012). Added to this, we now have online gaming via dedicated home consoles, personal computers, laptops, smartphones, tablets, and MP3 players. Given that there as many ways to play videogames offline as online, it makes greater conceptual sense to view IGD as a sub-type of gaming disorder rather than as a sub-type of IA (particularly—as argued above—because online gaming addicts are not addicted to the Internet, they are addicted to the gaming). The World Health Organization’s proposed diagnosis of Gaming Disorder in the beta draft of the (11th edition) International Classification of Diseases (with sub-varieties of online and offline gaming disorders) appears to be much more logical and well thought out than the description of Internet Gaming Disorder found in the DSM-5 (Saunders et al. 2017).

### The Context of Online Screen Time Is Far More Important than the Time Spent Online

Another key issue raised by Ryding and Kaye is the time spent gaming (often viewed as a key criterion of addictive gaming when played to excess) and the issue of online *“screen time”* more generally. While there is clearly a correlation between time spent playing and individuals that have IGD (Griffiths et al. 2016), the reverse is not necessarily the case. This is because the context is far more important than time when it comes to defining key characteristics of IGD and because there are many individuals who are excessive gamers but very few who would endorse five or more of the DSM-5’s criteria for IGD diagnosis (Griffiths 2010). Given that many of us spend hours a day online for work purposes (myself included) clearly demonstrates that *how the medium of the Internet is used* is much more important than the time spent in the medium when defining IA. Even when the excessive use of the Internet is work-based and causing long-term negatively detrimental effects on the individual, such individuals would be defined and conceptualized as work addicts rather than Internet addicts (Quinones and Griffiths 2017; Quinones et al. 2016).

### Researchers in the Online Addiction Field Can Learn a Lot from Those in the Problem Gambling Field

One of the major weaknesses in the IA field is that the majority of the data is self-report and based on non-representative epidemiological self-selected samples. However, there is an increasing amount of studies using neuroimaging techniques (see Pontes et al. (2017) for a recent review), a small number of studies using clinical samples of those undergoing treatment for IGD or IA (e.g., Beranuy et al. 2013). Many of the methodological weaknesses in the IA field are similar to those found in the gambling study fields a few decades ago. However, the growth of research into problem gambling has led to the collection of high-quality data from many different sources, and the IA field could learn a lot from the types of research and methodologies used by researchers in the gambling study field.

One of the major innovations in gambling is the access to (and the publishing of studies using) online behavioral tracking data. For example, over the past few years, Dr. Michael Auer and I have been given access to online gambling datasets from companies such as *win2day*, *Norsk Tipping*, and *Kindred Gaming*. We have used these data to develop new measures of *“gambling intensity”* based on actual gambling data (Auer and Griffiths 2014a, 2015a), evaluate the efficacy of responsible gambling tools such as limit setting (Auer and Griffiths 2013), pop-up messaging (Auer et al. 2014; Auer and Griffiths 2015b), and personalized feedback (Auer and Griffiths 2014b, 2015c, 2016), as well as directly comparing what gamblers say they have done online with what they have actually done online (Auer and Griffiths 2017a), and test classic psychological theories such as cognitive dissonance using subjective and objective measures from the same gamblers (Auer and Griffiths 2017b). Other researchers have used such data to identify behavioral markers of high-risk online gambling (Braverman and Shaffer 2012; Braverman et al. 2013; Gray et al. 2012) and to compare online gamblers who self-exclude with those that do not (Dragicevic et al. 2015).

In relation to IA and IGD, the use of behavioral tracking data may lead to innovative ways forward in the online addiction field, as well as new theoretical insights. This is because every click and transaction of every customer on every online site is automatically stored in a database and provides very high-data quality for those who know what to do with it. This is very much in contrast to traditional surveys where such data have to be gathered at high costs, with lots of manual work involved, and may be prone to error. Online video-gaming sites and social networking sites typically have (i) personal details provided by a user (e.g., name, email address, telephone number, address, gender,) and (ii) usage data collected automatically as the user clicks from one webpage to another (e.g., how users access the site, type of web browser used, time spent logged onto the site, what activities are engaged in, who they are engaging with,). If academics can form strategic collaborations with such commercial companies, they may be able to start identifying behavioral markers of excessive online users, gamers, and/or social networkers (that will include a small minority of problematic users). However, other types of research are also needed including longitudinal studies so that people can be tracked over time (either via behavioral tracking and/or surveys), and more detailed qualitative work (interviews and focus groups) with different player or user types, and those who seek treatment for their problematic use of online activities.

### Concluding Comments

The present paper summarized and extended some of the issues raised by Ryding and Kaye. In conclusion, it is argued that (i) Internet addiction (IA) is now a misnomer, (ii) IA and Internet Gaming Disorder (IGD) are two completely different constructs and that IGD is a sub-type of gaming disorder rather than a sub-type of IA, (iii) the time spent engaging in online activities is not a good criterion for assessing online addictions and that the context of use is a far more important criterion, and (iv) researchers working in the online addiction field can learn a lot from the problem gambling field in collecting robust data, and that one of the innovative ways forward could be to build strategic partnerships with commercial online companies to analyze their behavioral tracking data.
